# Culture-Related and Individual Differences in Regional Brain Volumes: A Cross-Cultural Voxel-Based Morphometry Study

**DOI:** 10.3389/fnhum.2019.00313

**Published:** 2019-09-10

**Authors:** Chih-Mao Huang, Robert Doole, Changwei W. Wu, Hsu-Wen Huang, Yi-Ping Chao

**Affiliations:** ^1^Department of Biological Science and Technology, National Chiao Tung University, Hsinchu, Taiwan; ^2^Cognitive Neuroscience Laboratory, Institute of Linguistics, Academia Sinica, Taipei, Taiwan; ^3^Center for Intelligent Drug Systems and Smart Bio-devices (IDS2B), National Chiao Tung University, Hsinchu, Taiwan; ^4^Graduate Institute of Mind, Brain and Consciousness, Taipei Medical University, Taipei, Taiwan; ^5^Brain and Consciousness Research Center, Taipei Medical University-Shuang Ho Hospital, Taipei, Taiwan; ^6^Department of Linguistics and Translation, City University of Hong Kong, Kowloon, Hong Kong; ^7^Graduate Institute of Biomedical Engineering, Chang Gung University, Taoyuan, Taiwan; ^8^Department of Computer Science and Information Engineering, Chang Gung University, Taoyuan, Taiwan; ^9^Department of Neurology, Chang Gung Memorial Hospital at Linkou, Taoyuan, Taiwan

**Keywords:** cultural differences, cultural values, independence-interdependence orientations, magnetic resonance imaging, voxel-based morphometry

## Abstract

Converging behavioral and functional neuroimaging evidence indicates that East Asian and Western individuals have different orientations for processing information that may stem from contrasting cultural values. In this cross-cultural magnetic resonance imaging (MRI) study, we used voxel-based morphometry (VBM) approach to investigate culture-related and individual differences of independent-interdependent orientation in structural brain volume between 57 Taiwanese and 56 Western participants. Each participant’s degree of endorsement of independent and interdependent cultural value was assessed by their self-report on the Singelis Self-Construal Scale (SCS). Behaviorally, Taiwanese rated higher SCS scores than Westerners in interdependent value and Westerners rated higher SCS scores than Taiwanese in independent value. The VBM results demonstrated that Western participants showed greater gray matter (GM) volume in the fronto-parietal network, whereas Taiwanese participants showed greater regional volume in temporal and occipital regions. Our findings provide supportive evidence that socio-cultural experiences of learned independent-interdependent orientations may play a role in regional brain volumes. However, strategic differences in cognition, genetic variation, and/or modulations of other environmental factors should also be considered to interpret such culture-related effects and potential individual differences.

## Introduction

There is a growing interest in how differences in culturally-based social and cognitive environments influence the way people construe themselves and perceive the visual world. A large literature of psychology and anthropology has provided innumerable demonstrations that there are subtle variations in the way people process information that appears to be a product of sociocultural experiences (Nisbett et al., 2001; Nisbett and Masuda, 2003; Han and Northoff, 2008; Kitayama and Uskul, 2011; Huang and Park, 2013). Although there are a variety of dimensions along which socio-cultural experiences would differ, one dimension that has received a large amount of attention involves the difference between independent culture (hypothesized to be predominant in Western cultures, such as North America) and interdependent culture (hypothesized to be predominant in East Asian cultures, including Taiwan, Japan, Korea, and China). Specifically, individuals with an independent, individualistic, and self-based focus of their sociocultural values may have a tendency to process focal and discrete objects of the environment (e.g., analytic processing), organize information *via* rules and categories, and emphasize personal agency and uniqueness. In contrast, individuals with an interdependent, collectivistic, and group-based focus of their sociocultural values are more sensitive to contextual information of the environment (i.e., holistic processing) and to relations among people and social harmony (Markus and Kitayama, 1991; Triandis, 1995; Hong et al., 2001; Nisbett et al., 2001; Oyserman et al., 2002; Nisbett and Masuda, 2003). Thus, far, systematic culture-related differences influenced by sociocultural values can be observed between East Asians and Westerners with respect to cognitive function, such as visual perception, memory, attention, and reasoning (Nisbett et al., 2001; Nisbett and Masuda, 2003; Goh and Park, 2009; Park and Huang, 2010; Schwartz et al., 2014; Na et al., 2017), as well as psychosocial processes such as relationality, social judgment, and self-concept (Markus and Kitayama, 1991; Han and Northoff, 2008; Kitayama and Uskul, 2011; Huang and Park, 2013).

Human neuroimaging studies have demonstrated that prolonged exposure to external experiences, practice, and training have a modulatory influence on behavior and brain structure. For example, Sluming et al. (2002) showed that symphony orchestra musicians have larger brain volume in left inferior frontal gyrus than control participants, possibly due to their expertise in musician-specific visuospatial performance (Sluming et al., 2002). In addition, Erickson et al. (2011) reported that older adults with 1-year of moderate-intensity exercise show increased volume of the hippocampus and improved memory function compared to a control group (Erickson et al., 2011). It is, therefore, no surprise that decades of exposure to a specific sociocultural system could shape or mold brain structure.

To date, only a few human neuroimaging studies have focused on exploring the brain structural differences between Eastern and Western cultures. In an early study, Zilles et al. (2001) conducted a magnetic resonance imaging (MRI) study to examine cultural differences in gross brain size and shape between Japanese and Europeans and reported that Japanese had relatively shorter but wider brains compared to Europeans (Zilles et al., 2001). Kochunov et al. (2003) compared the brain structure of Chinese-speaking East Asian and English-speaking Caucasian adults using MRI and concluded that greater frontal, temporal, and parietal regions shown in Chinese than Americans may stem from the linguistic characteristics of exercising Chinese (Kochunov et al., 2003). Similarly, Green et al. (2007) applied voxel-based morphometry (VBM) analysis to the brains of monolingual and multilingual speakers and demonstrated the greater frontal and temporal brain density in Chinese multilingual and European multilingual speakers relative to monolingual English speakers, suggesting an additional brain effort required for practicing tonal language (Green et al., 2007). In addition, Chee et al. (2011) examined a larger sample of structural brain images of non-Asian Americans and Singaporeans using VBM and cortical thickness measures. They demonstrated that, with well-matched cognitive function between two cultural groups, non-Asian Americans showed thicker cortical gray matter (GM) than Singaporeans in frontal, parietal, and temporal polymodal association regions, whereas Singaporeans revealed thicker left inferior temporal regions (Chee et al., 2011). More recently, Tang et al. (2018) compared 45 male Caucasian brain images selected from the Human Connectome Project (HCP) database (Van Essen et al., 2012) with 45 male Chinese recruited from the local community in China to investigate cultural/ethnic differences in brain volume and cortical thickness. They demonstrated that the male Chinese participants showed larger brain volume and cortical thickness in the temporal cortex and cingulate regions, whereas male Caucasians revealed greater brain volume and thicker cortical GM in the frontal and parietal regions (Tang et al., 2018). The discrepancy between these structural neuroimaging results suggests the presence of underlying mediators, related to volume and cortical thickness, particularly individual differences in socio-cultural values in the context of East Asian and Western nations.

Given the adaptive nature of individuals across East Asian and Western nations to endorse cultural values, there have been debates whether the differences in cognition and social behavior between independent culture and interdependent culture should be treated as two separate dimensions or a bipolar dimension (Oyserman et al., 2002; Brewer and Chen, 2007). Moreover, the cross-cultural psychological and neuroimaging data has suggested that nationality (e.g., United States or China) and/or cultural affiliation (e.g., American or Chinese) may not necessarily be reliable predictors of cultural values (Oyserman et al., 2002; Chiao et al., 2009). Therefore, in addition to the approach of directly contrasting East Asians and Westerners (Zilles et al., 2001; Kochunov et al., 2003; Green et al., 2007; Chee et al., 2011; Tang et al., 2018), recent structural neuroimaging studies administrated self-report measures to examine the influences of cultural values on brain structure when participants were recruited with identical nationality/culture. For example, Wang et al. (2017) collected structural brain images from a large sample of Chinese young adults to assess individual differences in the orientation of independence-interdependence cultural values. They administrated self-report questionnaires of the Self-Construal Scale (SCS, Singelis, 1994), Horizontal-vertical Individualism-Collectivism Scale, and computed factor scores of independence and interdependence *via* factor analysis to control for the response bias to affirm cultural values. The VBM results for the whole-brain analysis showed that the independence-interdependence score was related to greater GM volume in right dorsolateral prefrontal and right rostrolateral prefrontal regions that may be involved in self-related information processing (Wang et al., 2017). Similarly, Kitayama et al. (2017) performed a structural MRI study on 135 Japanese young adults while assessing their independent and interdependent self-construals to examine the relationship between cultural value and brain volume. Despite they found no significant correlations between independent self-construal and GM volume in Japanese brain, they demonstrated significant correlations between interdependent self-construal and brain volume of the bilateral orbitofrontal cortex (OFC), with Japanese individuals endorsing higher interdependent self-construals predicting reduced bilateral OFC volumes (Kitayama et al., 2017). In a follow-up study, Yu et al. (2018) further provide epigenetic evidence that such systematic cultural differences in the brain volume of OFC were moderated by genetic allele of the dopamine D4 receptor, suggesting an interactive pathway between biological system and socio-cultural values (Yu et al., 2018).

Here, we conducted a cross-cultural MRI study to not only compare brain volumes between East Asians and Westerners but also examine the associations between cultural orientation and regional brain structures at the individual level. Specifically, we examine whether regional brain structures differ between Taiwanese and Western healthy adults and how such culture-related effects vary as a function of independence-interdependence cultural orientations among individuals. We define individuals’ independence-interdependence cultural orientations based on participants’ self-report on the 30-item SCS (Singelis, 1994). The composite score of the SCS was computed and used as a continuous variable to conduct regression analyses to investigate the associations between cultural values and regional brain volume. Moreover, given that *post hoc* mediation analysis provides a theoretical way of investigating the role of intermediate variables which may play a role in the relationship between two other variables (Na et al., 2017; Fan et al., 2018), we applied this approach to examine whether cultural orientation play a role in explaining culture-related and individual differences in regional gray-matter volume. Based on prior neuroimaging works showing that individuals with a more independent focus are associated with increased brain volume in prefrontal cortex (PFC; Kitayama et al., 2017; Wang et al., 2017) and that Westerners had greater brain volume in fronto-parietal regions (Zilles et al., 2001; Kochunov et al., 2003; Green et al., 2007; Chee et al., 2011; Tang et al., 2018), we hypothesized that two cultural groups would show culture-related volumetric differences in fronto-parietal networks. Moreover, individuals with greater independent focus would show a greater gray-matter volume in fronto-parietal regions, regardless of their nationality and/or cultural affiliation.

## Materials and Methods

### Participants

A total sample of 113 right-handed university adults with no history of neurological or psychiatric disease participated in this cross-cultural MRI study. Fifty-seven participants were Taiwanese young adults (M = 23.7 years, SD = 2.55; 27 female and 30 male) who were born and raised in Taiwan. Fifty-six participants were Westerners^1^ (M = 24.0 years, SD = 2.98; 25 female and 31 male) who were born in Western countries (e.g., the United States, Canada, German, French, etc.) and are currently studying in Taipei, Taiwan. All Western participants were excluded if they spent more than 2 years in Asia. This study was approved by the National Taiwan University Institutional Review Board and all participants gave written informed consent prior to their participation. Group demographic information is reported in Table 1.

**Table 1:** Participants characteristics.

	Taiwanese (*N* = 57)			Westerners (*N* = 56)		
	Mean		SD	Mean		SD
Age (years)	23.67		2.55	24.00		2.98
Gender (M/F)		30/27			31/25
Education (years)	13.86		2.27	13.81		2.40
SCS*	−0.29		0.83	0.44		0.79
ICV (liters)	1.4836		0.1524	1.4676		0.1315

Abbreviations: M/F, Male/Female; SCS, Self-Construal Scale; ICV, global intracranial volume. *Significant main effects of culture at *p* < 0.001.

### Questionnaire of Independence-Interdependence Orientations

There is cultural psychological evidence demonstrating that nationality (e.g., United States) and cultural affiliation (e.g., American) may not necessarily be reliable predictors of independent and interdependent cultural values (Oyserman et al., 2002; Chiao et al., 2009). Therefore, we administrated the SCS (Singelis, 1994) to assess how strongly participants subscribe to cultural values of independence and interdependence. After being scanned in an MRI, each participant completed the 30-item version of the SCS (Singelis, 1994) to measure individual variations in independence-interdependence orientations with 15 independent items, such as “I enjoy being unique and different from others in many respects,” and 15 interdependent items, such as “I do my own thing regardless of what others think.” Each item is rated on a scale from 1 (Strongly Disagree) to 7 (Strongly Agree). The alpha coefficients were calculated to assess the reliabilities for the two subscales of SCS for each cultural group. In this cross-cultural study, the alpha coefficients for the independence and interdependence subscales of SCS were 0.72 and 0.80 for Taiwanese group, respectively, and 0.73 and 0.67 for Western group, respectively.

To calculate each participant’s SCS score, mean agreement for 15 independent and 15 interdependent items in SCS was computed as a composite score for each participant with the scoring algorithm: SCS score = [(mean of agreement for independent items) − (mean of agreement for interdependent items)] (Chiao et al., 2009). The scoring algorithm results in positive values for predominantly independent cultural value (SCS score > 0) and negative values for predominantly interdependent cultural value (SCS < 0).

All participants’ SCS scores were then used as a continuous variable to conduct regression analyses on regional brain volume to investigate the associations between individual differences in independent-interdependent cultural values and the magnitude of volumetric differences of brain structure.

### Imaging Acquisition

A 3-Tesla MRI scanner (Skyra, Siemens, Erlangen, Germany) with a 20-channel head coil at the Taiwan Mind and Brain Imaging Center in National Chengchi University, Taipei, Taiwan was used for all MRI data acquisition. High-resolution, three-dimensional, sagittal-oriented, T1-weighted anatomical images were collected using a three-dimensional (3D) ultrafast magnetization-prepared rapid acquisition with gradient echo (MPRAGE) imaging sequence with the following parameters: FOV: 256 × 256 mm^2^, resolution: 256 × 256, slice thickness = 1 mm, slice number = 192, TR/TE = 2530/3.3 ms, TI = 1,100 ms, and flip angle = 7°.

### Voxel-Based Morphometry Processing

All MRI data were preprocessed and analyzed in Statistical Parametric Mapping (SPM12^2^) implemented in MATLAB R2014a (The Mathworks inc., Natick, MA, USA). The Voxel Based Morphometry (VBM) analyses were employed following the Diffeomorphic Anatomical Registration Through Exponentiated Lie algebra (DARTEL) approach (Ashburner, 2007). The VBM-DARTEL processing included the following steps: (1) the T1 structural images were segmented into white matter (WM), GM, and cerebrospinal fluid (CSF) tissue probability maps (WM-TPM, GM-TPM and CSF-TPM) using the standard unified segmentation model in SPM12; (2) then three TPMs of each subject were co-registered with a standard TPM template implemented in SPM12, respectively; (3) the three types of co-registered TPMs of all Taiwan and Foreign subjects (total subject number = 113) entered into a nonlinear image co-registration procedure using diffeomorphic anatomical registration through DARTEL technique, which involves iteratively matching all the selected images to a template generated from their own mean (Ashburner and Friston, 2005); and (4) the individual segmented GM-TPM were then warped to a study-specific template in the standard MNI space using the resulting flow fields created by DARTEL with resampling voxel size of 1 mm isotropic and spatially smoothed with 8-mm full-width at half maximum Gaussian kernel.

### Statistical Analyses

Two major statistical analyses were performed in this cross-cultural VBM study. To examine culture-related differences in regional gray-matter volumes, the voxel-wise generalized linear modeling (GLM) was applied within the MNI space mask across the whole brain. Voxel-wise parametric statistical tests, which directly compare the smoothed MRI images from two cultural groups, was then performed to identify specific regions in which GM areas showed significantly volumetric differences between Taiwanese and Westerners participants, controlling for global intracranial volume (ICV)^3^, age, and gender. An absolute threshold mask of 0.2 was applied to avoid edge effects within the boundary of GM areas. Statistical maps were assessed by applying the Threshold-Free Cluster Enhancement methods (TFCE^4^, version 174) to all the models used in this study. The TFCE method is a non-parametric permutation-based approach that requires no arbitrary definition of voxel-wise or cluster thresholds on neuroimaging data (Smith and Nichols, 2009). All TFCE-based analyses were performed with default parameters of 5,000 permutations, *E* = 0.5 and *H* = 2. We consequently established a conservative family-wise error (FWE)-corrected threshold of *p* < 0.05.

To investigate the relationship between individual differences in independence-interdependence orientations and cross-culture gray-matter volume, we then performed the multiple regression analyses by using the TFCE to identify specific brain regions in which gray-matter correlated with the SCS scores and the two subscale scores (i.e., independence and interdependence subscales of SCS) separately across the whole brain, controlling for global ICV, age, and gender. Statistical maps were assessed at FWE-corrected threshold of *p* < 0.05.

## Results

### Demographic Results

Table 1 presents the participant characteristics of 57 Taiwanese adults and 56 Westerners in the study. The two cultural groups showed significant differences in orientation of independent-interdependent cultural values, with Westerners rated higher SCS scores than Taiwanese on independent items and Taiwanese participants rated higher SCS scores than Westerners on interdependent items (Taiwanese: mean = −0.29, SD = 0.83; Westerners: mean = 0.44, SD = 0.79; *p* = 0.00000523). There were no significant differences in age (*p* = 0.50) and educational levels (*p* = 0.91) between Taiwanese and Westerners.

### Voxel-Based Morphometry Results

**Figure 1:**
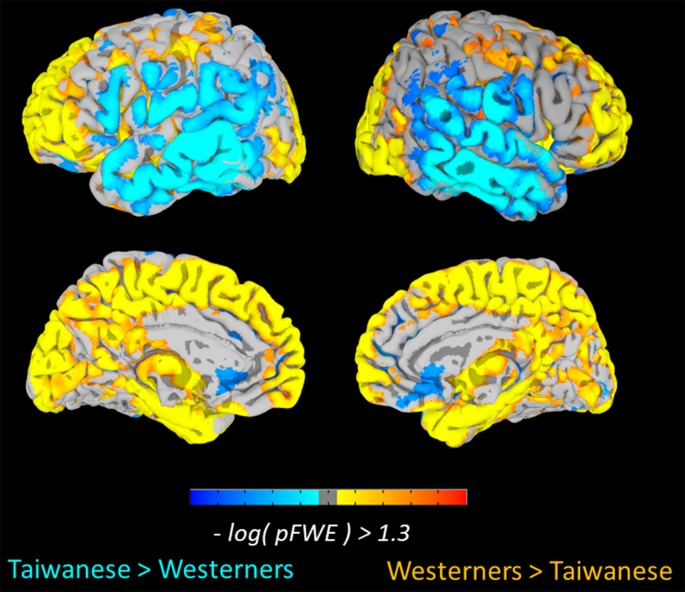
Culture-related differences in the regional brain volume. The voxel-based morphometry (VBM) analysis identified cultural differences in gray matter (GM) volumes between Taiwanese and Westerner groups [Threshold-Free Cluster Enhancement (TFCE), family-wise error (FWE) *p* < 0.05].

To examine culture-related differences in regional gray-matter volume, we first performed a whole-brain VBM analysis to identify the significant differences in the gray-matter volume of brain regions between two cultural groups using TFCE method with FWE-corrected threshold of *p* < 0.05. The Taiwanese participants showed greater regional gray-matter volume in left and right middle temporal gyrus, right inferior temporal gyrus, left middle occipital gyrus, right calcarine sulcus, and right superior occipital gyrus. In contrast, the Western participants showed greater volume in frontal-parietal network, including left superior medial frontal gyrus (medial PFC), left superior frontal gyrus (superior PFC), and left postcentral gyrus. Significant culture-related differences in brain regions between Taiwanese and Western groups are illustrated in Figure 1 and Table 2.

**Table 2:** Brain regions showing culture-related differences in gray-matter volumes.

Cluster size [voxels]	L/R	Brain region	MNI Coordinates			pFWE
			X	Y	Z	
**Taiwanese > Westerners**				
19961	L	Middle temporal gyrus	−70	−13	−17	0.004
	L	Middle temporal gyrus	−70	−17	−6	0.004
	L	Middle temporal gyrus	−71	−27	−5	0.004
10989	R	Middle temporal gyrus	71	−28	−5	0.004
	R	Inferior temporal gyrus	70	−31	−19	0.004
	R	Middle temporal gyrus	71	−25	−13	0.004
3506	L	Middle occipital gyrus	−21	−60	33	0.004
	L	Middle occipital gyrus	−26	−66	39	0.004
	L	Middle occipital gyrus	−31	−74	27	0.004
1117	R	Calcarine sulcus	21	−88	1	0.004
2216	R	Caudate	16	−10	17	0.004
333	R	Superior occipital gyrus	32	−63	39	0.004
155	R	Calcarine sulcus	16	−76	8	0.004
**Westerners > Taiwanese**						
396757	L	Superior medial frontal gyrus	−8	68	17	0.001
	L	Superior frontal gyrus	−7	71	8	0.001
	L	Superior medial frontal gyrus	−7	65	25	0.001
722	R	Lingual gyrus	22	−52	−10	0.001
649	L	Postcentral gyrus	−55	−12	43	0.001
233	L	Middle temporal gyrus	−59	−69	4	0.001
68	R	Superior frontal gyrus				

*Abbreviations: L/F, left/right hemisphere; pFWE, family-wise error (FWE)-corrected threshold *p*-value. Threshold-Free Cluster Enhancement (TFCE), FWE *p* < 0.05*.

To examine whether individual variations in independence-interdependence orientations modulates volumetric differences of regional brains, we performed the whole-brain multiple regression analyses using TFCE method with FWE-corrected threshold of *p* < 0.05 to identify specific brain regions whose volume is predicted by the SCS scores, adjusted for global ICV, age, and gender. However, no cluster was found with a significant positive or negative correlation with regional gray-matter volume for the SCS score, independence subscale score and interdependence subscale score. Additionally, when an extent-threshold (Wang et al., 2017) or a height-threshold (Kitayama et al., 2017) based on random field theory were applied, no cluster was found with a significant correlation with regional gray-matter volume for the SCS score, independence subscale score and interdependence subscale score.

## Discussion

In this cross-cultural MRI study, we not only compare brain volumes between East Asians and Westerners but also examine the association between cultural orientation and brain volumes at the individual level. Our VBM results clearly demonstrated culture-related structural differences in regional brain volume between two cultural groups, with larger occipital and temporal cortices for individuals raised in an East Asian culture (i.e., Taiwanese) and larger frontal and parietal regions, and cerebellum for individuals from a Western culture. Taiwanese participants showed significantly higher SCS scores than Westerners in interdependent cultural value, whereas Westerners rated higher SCS scores than Taiwanese in independent cultural value. However, individuals with the spectrum of independent-interdependent orientation did not show positive or negative significant association between regional gray-matter volume and the SCS scores. Our findings provide neuroimaging evidence that culture-related experience may influence regional brain structures.

Culture-related structural differences in regional brain volume observed in our results between Taiwanese and Westerners are consistent with previous structural neuroimaging findings using various data analysis methods, including VBM, cortical thickness measures, and pattern classification approaches (Chee et al., 2011; Tang et al., 2018). Given the notion that sustained exposure to external experiences, perceptual and cognitive practices affect behavior, neural function and brain structure, the VBM results suggest an analytic-holistic dichotomy for culture variations in visual perceptual process. Taiwanese (vs. Westerners) showed greater gray-matter volume in visual regions (including calcarine sulcus, inferior and middle temporal gyrus, and superior and middle occipital regions), which may reflect their holistic information-processing bias for frequently detecting and integrating contextual associations between objects and backgrounds in the perceptual world from their interdependent culture (Gutchess et al., 2006; Goh and Park, 2009; Jenkins et al., 2010; Park and Huang, 2010). For example, Goh et al. (2009) reported eye-movement evidence for more attention to context in East Asians, with shorter fixation durations that frequently alternated between objects and backgrounds, whereas Westerner showed longer duration to fixating on the focal objects (Goh et al., 2009). Alternatively, such culture-related structural differences in visual cortices may be driven by visual complexity of perceptual environment. For example, Miyamoto et al. (2006) demonstrated that the physical environment of Japan is more ambiguous than that of the United States and that it contains a greater variety of visual elements. Therefore, prolonged exposure to visually complex environments would induce more cognitive effort and perceptual practice for Japanese to process contextual information of the environment with the result that they recruit more neural resources (Miyamoto et al., 2006; Chee et al., 2011).

Moreover, the similar results that Western participants showed greater volume in frontal regions, including left medial frontal gyrus and left superior frontal gyrus compared with Taiwanese participants have been reported in previous structural neuroimaging studies (Chee et al., 2011; Tang et al., 2018). Given the evidence that these prefrontal regions are associated with the cognitive and affective processes of decision making, conflict monitoring, error detection, and executive control, the increased volume in these areas could conceivably be due to the increased focus on reasoning, analytic process, and independent thinking which has been emphasized by a Western sociocultural environment and educational settings (Chee et al., 2011). Alternatively, increased prefrontal volume in medial frontal gyrus for Westerners may reflect the prolonged practice involved in processing self-generated information and self-referential cognition due to the emphasis on personal agency and uniqueness from independent cultural values (Markus and Kitayama, 1991; Nisbett and Masuda, 2003; Wang et al., 2017). For example, in a recent meta-analysis of functional neuroimaging studies, Han and Ma (2014) reported culture-related differences in brain activation underlying social and non-social processes. They demonstrated that Western participants showed increased brain activation in the neural network related to self-reflection and emotional responses whereas East Asians showed greater brain activity in the brain areas sensitive to the process of mentalizing and emotional regulation during social cognitive/affective processes (Han and Ma, 2014).

The effect of culture reported in this study, however, is dissimilar to some of the previous studies (Kochunov et al., 2003; Green et al., 2007). Kochunov et al. (2003) directly compared the structural MRI images of Chinese-speaking East Asian and English-speaking American adults and reported the greater frontal, temporal, and parietal regions for Chinese than Americans (Kochunov et al., 2003). Similarly, Green et al. (2007) directly contrasted the structural MRI images of monolingual English and multilingual Chinese/European speakers and demonstrated the greater frontal and temporal brain density in multilingual speakers relative to monolingual speakers (Green et al., 2007). Both of the studies suggested that such group effects may reflect additional brain resources for processing tonal distinction and exercising linguistic characteristics of Chinese language. In contrast to these studies focusing on group differences in language experiences, the current cross-cultural study systematically examined the influences of independent-interdependent orientation at the group level as well as at the individual level with the fact that all participants were at least bilingual or multilingual in English and their native languages (Chinese, French, German, Spanish, etc.). Moreover, given the recent neuroimaging findings suggest the universal language network underlying Chinese and alphabetic languages in orthographic, phonological, and semantic processing (Wu et al., 2012; Rueckl et al., 2015), our findings demonstrated the culture-related differences of independent-interdependent cultural orientation in structural brain volume and such effect influenced by individual’s language experiences is minimal.

There is no positive or negative statistically significant associations between regional gray-matter volume and the SCS scores when TFCE method with FWE-corrected threshold of *p* < 0.05 was applied in this study. We employed the TFCE method to correct for multiple comparisons, which is different from the previous studies using either an extent-threshold (Wang et al., 2017) or a height-threshold (Kitayama et al., 2017) based on random field theory. In order to compare the current study with the two studies, a voxel-wise threshold of uncorrected *p* < 0.005 and cluster-extent threshold of FWE *p* < 0.05 used in Wang et al. (2017) and a voxel-wise threshold of FWE *p* < 0.05 used in Kitayama et al. (2017) were further applied to the current data. However, we failed to find evidence for individual differences of SCS scores, independence subscale score and interdependence subscale score in regional brain volume. Therefore, our results are inconsistent with previous neuroimaging studies administrating self-report measures to examine the influences of cultural values on brain structure when participants were recruited with identical nationality/culture (Kitayama et al., 2017; Wang et al., 2017; Yu et al., 2018). Given the fact that the current study recruited participants from two cultural groups with identical MRI machines and acquisition protocols in East Asian area (i.e., Taiwan), and behaviorally, Taiwanese participants showed significantly higher SCS scores than Westerners in interdependent cultural value whereas Westerners rated higher SCS scores than Taiwanese in independent cultural value, our results suggest that individual differences in spectrum of independence-interdependence orientation may not the only factor to sculpt the neural correlates of culture-related variations in regional brain volume. Taken together, the previous studies showed considerable evidence for individual variations of socio-cultural values and regional brain volume, but more work is needed to understand the variables controlling cultural differences in independence-interdependence orientation to brain structure.

Despite the similarities between the previous and the current MRI studies, however, we should note that there are several discrepancies between studies that may be driven by other unknown genetic diversity, environmental biases, and/or gene-environment interaction across multiple time scales (Chee et al., 2011; Chiao et al., 2013; Yu et al., 2018). Given the evidence that cultural differences in the specific brain regions and social behaviors were moderated by genetic allele in young adults (Chiao and Blizinsky, 2010; Yu et al., 2018), future works along this line may provide more integrative framework for understanding the interactive nature between biological system, psychological functions, adaptive behaviors, and socio-cultural values (Park and Gutchess, 2002, 2006; Kitayama and Uskul, 2011; Chiao et al., 2013; Han et al., 2013; Kitayama et al., 2017). Moreover, cultures are not defined by only the individual member’s affiliation and/or nationality along an independence-interdependence spectrum. Other dimensions such as “power distance,” “masculinity vs. femininity,” and “uncertainty avoidance” as well as “local subdivisions of geography (e.g., United States south vs. north)” are also defined as culture from different perspectives (Hofstede, 1978, 1984; Hofstede and Bond, 1984; Gutchess and Goh, 2013; Doole et al., 2015). Our results clearly demonstrated culture-related differences in regional GM volume, thus, it is reasonable to posit that other dimensions of cultural differences would also result in the regional differences in brain volume and/or cortical thickness. Future studies will establish more mechanistic links between various domains of culture, human behavior, and brain structure, as well as how brain structure explains cultural differences in behavior.

## Ethics Statement

This study was approved by the National Taiwan University Institutional Review Board and all participants gave informed consent prior to their participations.

## Author Contributions

C-MH, RD, CW, H-WH and Y-PC contributed to the conception and experiment design. C-MH, RD, CW, H-WH and Y-PC contributed to preparation and revision of the manuscript. C-MH, CW and Y-PC contributed to data analysis. All authors reviewed and approved the manuscript.

## Conflict of Interest Statement

The authors declare that the research was conducted in the absence of any commercial or financial relationships that could be construed as a potential conflict of interest.

## Footnotes

^1^Nationalities of Western participants: 27 participants from Europe (48.2%), nine participants from U.S. (16.1%), five participants from Canada (8.9%), 13 participants from Central America (23.2%), and two participants from Australia (3.6%). All nationality showed positive SCS scores in average.

^2^https://www.fil.ion.ucl.ac.uk/spm/software/spm12

^3^Global intracranial volume (ICV) is calculated by a sum of gray matter volume, white matter volume, and cerebrospinal fluid for each individual.

^4^http://dbm.neuro.uni-jena.de/tfce/
